# Slippage of degenerate primers can cause variation in amplicon length

**DOI:** 10.1038/s41598-018-29364-z

**Published:** 2018-07-20

**Authors:** Vasco Elbrecht, Paul D. N. Hebert, Dirk Steinke

**Affiliations:** 1 Centre for Biodiversity Genomics, University of Guelph, 50 Stone Road East, Guelph, Ontario N1G 2W1 Canada; 2 Department of Integrative Biology, University of Guelph, 50 Stone Road East, Guelph, Ontario N1G 2W1 Canada

## Abstract

It is well understood that homopolymer regions should be avoided for primer binding to prevent off-target amplification. However, in metabarcoding, it is often difficult to avoid primer degeneracy in order to maximize taxa detection. We here investigate primer binding specificity using different primer sets from several invertebrate metabarcoding studies. Our results indicate that primers frequently bound 1-2 bp upstream in taxa where a homopolymer region was present in the amplification direction. Primer binding 1 bp downstream was observed less frequently. This primer slippage leads to taxon-specific length variation in amplicons and subsequent length variation in recovered sequences. Some widely used primer sets were severely affected by this bias, while others were not. While this variation will only have small impacts on the designation of Operational Taxonomic Units (OTUs) by clustering algorithms that ignore terminal gaps, primer sets employed in metabarcoding projects should be evaluated for their sensitivity to slippage. Moreover, steps should be taken to reduce slippage by improving protocols for primer design. For example, the flanking region adjacent to the 3′ end of the primer is not considered by current primer development software although GC clamps in this position could mitigate slippage.

## Introduction

Metabarcoding permits the rapid assessment of biodiversity^1^ using amplicon-based high-throughput sequencing^2^. For metazoans, a segment of the cytochrome *c* oxidase subunit I (COI) gene is used^3^ as it offers species-level resolution coupled with access to extensive reference data^4^. However, sequence variability in this gene region makes primer design difficult, especially when analyzing bulk samples that include a broad array of taxa^5^. Mismatches between primer and template DNA can lead to substantial primer bias, causing some taxa to remain undetected^6,7^. Although ribosomal markers provide more conserved primer binding sites^8^, well designed COI primers can match or exceed the performance of ribosomal markers^9,10^.

A key component of successful COI metabarcoding primers is primer degeneracy to allow matching at variable binding sites^11^. Tools such as PrimerMiner support the automated download and processing of reference sequence data for the taxonomic group(s) targeted for analysis^12^. Sequence alignments built from such datasets help to identify suitable primer binding sites. Matching to variable binding sites is optimized by inserting degenerate bases into the primer sequence. However, high degeneracy increases the chance that primers will also bind to non-target regions^13^. While sequences from non-target regions can be filtered out bioinformatically or by size selection of PCR products (assuming different amplicon lengths), such filtration can reduce the yield of target fragments. Thus, primer degeneracy is a tradeoff between maximizing taxon recovery^7,11^ and primer specificity. In a previous study, we observed length variation among sequences recovered from most primer combinations (Fig. S6 in^11^), but did not investigate the mechanisms underlying this variation or the extent of variation in this effect at a species level.

In this study we analyze binding specificity for five primer sets in studies on mock assemblages of freshwater and marine macroinvertebrates^11,14,15^. These datasets were chosen because haplotype sequences for most specimens were known, allowing precise determination of primer binding behavior on both the targeted binding regions and flanking areas.

## Material and Methods

To investigate the specificity of primer binding, we analyzed five different primer sets used for metabarcoding of mock communities with known composition^11,14,15^. COI sequences spanning the Folmer region^3,16^ were available for most taxa which allowed the analysis of potential length variation in amplicons generated by each primer set and specimen at a haplotype level. Table 1 describes the primer combinations analyzed.

**Table 1:** The specificity of binding to different template strands for three forward and four reverse primers. The performance of each primer was examined for the ten most abundant taxa in each PCR reaction (for which the template sequences were known).

Primer combination	Primer tested	Length variation	Proportion with expected length (±SD)	t-test (p value)	Fig.	Data set
P5_BF1_0 + P7_BR1_4	BF1	−1 to 2 bp for some taxa	80.42 (±18.94)	0.003	Fig. 1A	(Elbrecht & Leese^11^)
	BR1	No variation	99.44 (±0.04)	NA	Fig. S1A	
P5_BF2_0 + P7_BR1_4	BF2	−1 to 2 bp for some taxa, +1 for all taxa	62.14 (±14.07)	NA	Fig. 1B	(Elbrecht & Leese^11^)
	BR1	No variation	99.45 (±0.04)	NA	Fig. S1B	
P5_fwhF1_3 + P7_fwhR1_1	fwhR1	+1 for all taxa	96.24 (±1.22)	NA	Fig. S1C	(Vamos *et al*.^14^)
P5_fwhR2_2 + P7_fwhF2_3	fwhR2	No variation	99.33 (±0.05)	NA	Fig. S1E	(Vamos *et al*.^14^)
	fwhF2	−1 to 2 bp for some taxa	82.23 (±22.05)	0.003	Fig. S1D	(Vamos *et al*.^14^)
mlCOIintF + jgHCO2198, complete run 1	mlCOIintF	−1 to 2 bp for some taxa	70.08 (±29.93)	0.003	Fig. S1F	(Leray & Knowlton^15^)

The exact primer length distribution and number of sequences used for this analysis are also provided in Table S1. For primers where no length variation was observed or for primers where all taxa showed length variation, no t-test could be applied (NA) due to the lack of groups (slippage vs no slippage).

The datasets were retrieved from the NCBI Sequence Read Archive and demultiplexed using the JAMP v0.34 pipeline (github.com/VascoElbrecht/JAMP)^11,14^. Only sequences of the randomly selected mock community “B” were analyzed from Elbrecht & Leese 2015. Sequence data from a study that examined marine macroinvertebrates^15^ were downloaded from figshare. The results from sequencing run 1 were used without demultiplexing to ensure sufficient sequencing depth. Raw sequences were paired end merged using Usearch v10.0.240 (fastq_mergepairs -fastq_pctid 90 -fastq_maxdiffs 999 -fastq_trunctail 0^17^), and imported into Geneious 11.0.4^18^. In bioinformatic analysis of metabarcoding data sets, sequences are often clustered by similarity into Operational Taxonomic Units (OTUs), to reduce data complexity. Based on OTU tables from the original studies, the ten most abundant OTUs for each primer combination were selected for analysis to ensure sufficient sequencing depth and to reduce stochastic effects^15^. Sequences from sample B and from the marine mock sample were mapped against the known haplotype sequence for each selected taxon (lowest sensitivity, a 100% match, and zero gaps in the sequence, haplotypes from Script S2 in^11^). Flanking regions in the sequence alignment were extracted for each taxon, and the length distribution of each primer sequence was determined. A few sequences (no more than three per taxon) were much longer than expected, likely due to sequencing artifacts, and were therefore excluded from further analysis, which still included several thousand sequences per taxon (Table S1). A t-test was used for each primer to differentiate between OTUs where 10% or more reads were affected by length variation and those that were unaffected. All R scripts used are available as supporting information (Scripts S1).

### Data Accessibility statement

Raw sequence used in this study data is available in the NCBI Sequence Read Archive under the accession numbers SRR5295658 and SRR5295659 (fwh1 primer set), SRX1619153 (BF/BR primer set), and figshare for the mlCOIintF/jgHCO primer set (R1 direction: 10.6084/m9.figshare.4039821.v1, R2 direction: 10.6084/m9.figshare.4039860.v1). The scripts used in this study are available as supporting information (Scripts S1). All sequence data extracted from the primer binding regions have also been uploaded to Dryad (10.5061/dryad.nk81st2).

## Results

**Figure 1:**
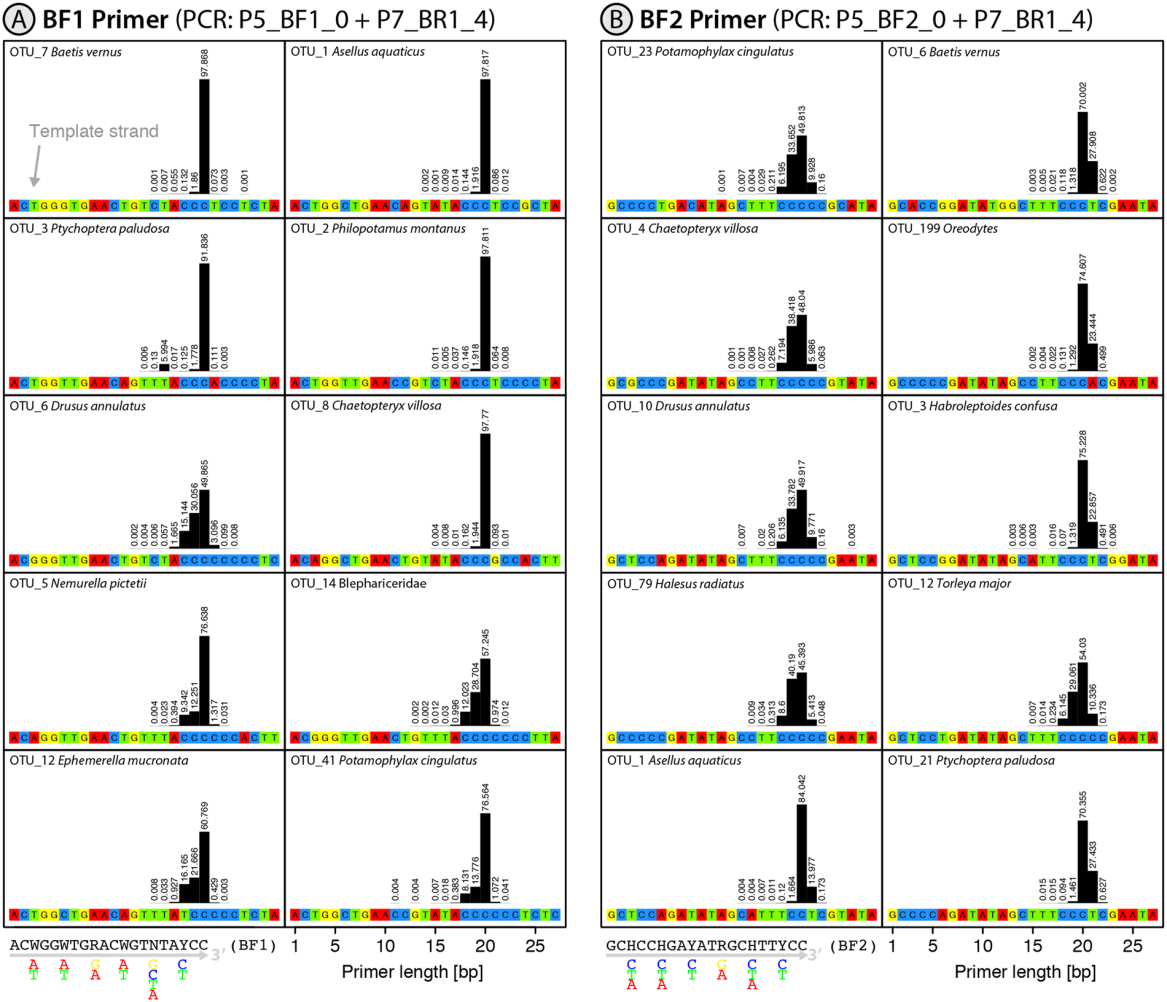
Plot showing the primer binding sites and bar plots depicting the length distribution of binding primers for the 10 most abundant OTUs in the mock sample B (sequence data from^11^. The sample was amplified with the N5_BF1_0 + N7_BR1_4 and N5_BF2_0 + N7_BR1_4 primer set, and the length distribution of the incorporated primers is shown for both the BF1 and BF2 primer (**A**,**B**). Relative abundance is given as a percentage above each bar.

Two of the three reverse primers (BR1, fwhR2) were not associated with length variation (>99% sequences had the expected length), but the other reverse and all four forward primers showed length variation (Table 1). A 1 bp insertion was present at the 3′ end of some (<10%) amplicons generated by the fwhR1 and the BF2 primers, (Figs 1B and S1C). Importantly, the 3′ end of the fwhR1 primer binds to a homopolymer region with up to six cytosines in some species while the BF2 primer targets a low complexity region of cytosine and thymine. In those cases where taxa amplified with the BF2 primer were unaffected by deletions, some sequences were affected by 1 bp insertions (Fig. 1B). Many of the sequences retrieved with the four forward primers (BF1, BF2, fwhF2 and mlCOIintF) were 1-2 bp shorter than expected (Figs 1 and S1C,D). The incidence of these truncated sequences varied among primers and with the nature of templates, with their frequency rising when a low diversity cytosine primer binding region extended in the direction of elongation. This effect was particularly dramatic for some taxa amplified with the mlCOIintF primer. For example, 80% of the sequences were shorter than expected for OTU_92 where the primer bound to a homopolymer region spanning seven cytosines (Fig. S1F). Interestingly, in taxa where this low diversity region was directly followed by a set of different nucleotides (e.g. a poly C region followed by A, T or G), <2% of the sequences were affected by deletions (Figs 1 and S1D,F). There was significantly more length variation between OTUs where binding sites were followed by low diversity regions than those binding sites that were flanked by high diversity variation for all tested primers (p = 0.003, t-test, Table 1). Some primers, such as BF2, were associated with both insertions and deletions (Fig. 1A). In a few cases, larger changes in sequence length were detected, apparently linked to compositional variation in the primer binding site. For example, OTU_3 possessed a tandem repeat (ACCC) within the primer binding region and, when it was amplified with BF1, about 6% of the sequences possessed a 4 bp deletion in the amplicon as primer sequences were only 16 bp long instead of 20 bp.

## Discussion

This study describes length variation created when degenerate primers bind to low diversity regions of their target template. This length variation does not reflect the presence of an indel in the primer or the template, but rather results from the primer binding 1-2 bp downstream or upstream from its expected site. In such situations, amplicons are 1-2 bp longer or shorter than expected once primers are trimmed during bioinformatics processing. The fact that primer sequences were successfully trimmed from >99% of the reads in each sample^11^ indicates that this variation reflects primer slippage rather than indels in the primer itself. Additionally, we detected a taxon-specific slippage effect in datasets from several independent studies that used different primer sets, making it unlikely this effect is caused by flaws in oligo synthesis. While we previously described primer-dependent length variation resulting from metabarcoding samples amplified with BF1/BF2^11^, the present study demonstrates this phenomenon for a wider range of primer sets using individual COI barcoded specimens. The overall results indicate that when the 3′ end of a primer binds to a low diversity region, the primer often also binds 1-2 bp away from its target binding region. We argue this process is influenced by primer degeneracy, by the composition of the template DNA, and by the length of the low diversity region in the template DNA, being most prominent when it exceeds the length in the primer binding region.

**Figure 2:**
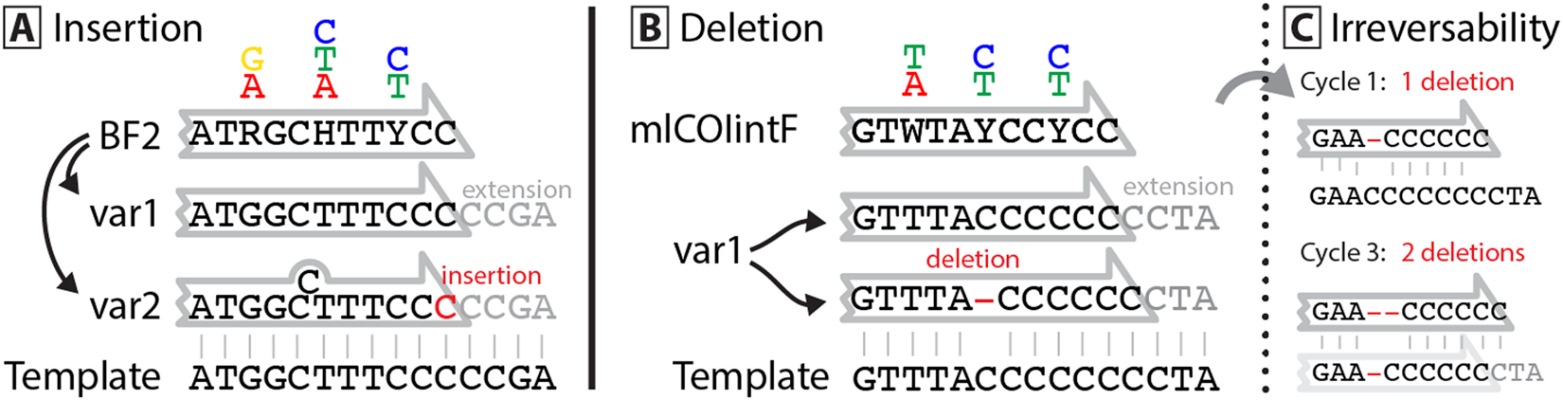
Proposed mechanism of primer slippage in binding regions with low diversity. Primer and template DNA are depicted with black letters, while nucleotides added during PCR are indicated by grey letters with insertions and deletions highlighted in red. (**A**) Highly degenerate primers include many different primer versions (e.g. var1 & var2). These can slip “backwards” in low diversity binding regions, as the 3′ primer tip can also match 1 bp upstream, leading to the incorporation of an additional base (shown here at the end of the BF2 primer). (**B**) Slippage in the forward direction is more common and follows a similar mechanism. The primer binds one position upstream which leads to the deletion of one nucleotide. When homopolymer regions are present at a primer binding site, forward slippage is much commoner than backward slippage. This effect is likely caused by the incorporation of primers throughout the PCR cycles (**C**), which can easily slip forward, but then shorten the homopolymer region providing less room for primers to bind and slip backward. If so, the extent of primer slippage should be PCR cycle dependent.

If primer slippage occurs, it usually involves a homopolymer region (e.g. CCCC) at the 3′ end and leads to the deletion of 1-2 bases. Insertions were less common and were limited to single base inserts in the primer sets examined in this study. Figure 2 depicts how these indels are likely caused through off-target primer binding. Analysis of variation in the incidence of these events among taxa indicated that forward primer slippage only occurred when a homopolymer region extended in the extension direction of the primer. This constraint means that primer slippage is highly template dependent with marked differences among species or even between haplotypes of a species. The explanation for this pattern is clear - the primer is prevented from binding upstream if the homopolymer region is interrupted by different nucleotides, preventing forward slippage. This also means that primer slippage can be prevented by targeting regions with higher diversity, or by providing two different base pairs at the (usually conserved) 3′ end (e.g. a GC clamp). For example, the BR1 primer binds in regions with up to a 4 bp homopolymer of cytosine, but it does not show signs of primer slippage because of the GC at its end and the absence of another cytosine flanking the primer binding region. In cases where the DNA template shows similar repetitive patterns, slippage of more than 2 bp is possible, e.g. OTU_3 amplified with the BF1 primer (Fig. 1A). In cases where the homopolymer region does not extend beyond the primer binding site, slippage can still occur in the opposite direction, leading to single base insertions as evidenced by both the fwhR1 and BF2 primers. The BF2 primer is particularly affected by insertions as it can bind to a poly-thymine/cytosine region, linked by a degenerate mixed base (Y = T or C).

In general, when primer slippage occurs, it leads to deletions rather than insertions, likely reflecting the irreversible nature of primer shifts. For example, if a primer is successfully incorporated and amplified one bp upstream of the usual binding site, it will shorten the homopolymer, making all successively amplified fragments shorter as well, or even leading to further forward shifts (Fig. 2C).

These issues have important implications for both primer design and for the bioinformatics analysis of sequence data. While it is generally recommended to avoid homopolymer regions in binding sites^19^, the variability of the COI barcoding fragment^5,20^, and the high degeneracy needed to reduce primer bias^6,7^, places strong constraints on primer design. Nevertheless, metabarcoding primers should be designed which bind to two different nucleotides at the 3′ end to reduce the chance of primer slippage. Further, because primer slippage events are highly template specific, the sequence attributes of both the primer binding region and its flanking regions should be considered. To our knowledge, software currently employed for primer design only considers the nucleotide composition of the targeted binding site, and ignores the flanking region in the extension direction. Therefore, we recommend evaluating that all primers used for metabarcoding analysis be tested for their susceptibility to slippage. Our study clearly shows that commonly used or recommended metabarcoding primers such as e.g. mlCOIintF^21^ or BF2^11^ are susceptible to substantial primer slippage.

Primer slippage can lead to a large proportion of sequences being a few bp longer or, more likely, shorter than expected. As this effect is highly template specific and differs between taxa, it can introduce substantial biases during bioinformatic processing. It can skew the representation of certain species or haplotypes, especially if a metabarcoding dataset is filtered to an exact amplicon length. If, on the other hand, sequences of slightly different length are included in the analysis, they could introduce a substantial bias by generating false OTUs if terminal gaps are counted as differences^22^. Thus, when analyzing metabarcoding data, it is essential to know if a primer set is sensitive to slippage, and if the results generated by the clustering algorithm are impacted by such variation. It is fairly easy to test for primer slippage by examining patterns of length variability in the amplicons and their location. If more than 10% of the sequences are 1-2 bp shorter than expected after primer trimming and the length variation is concentrated near the ends of the sequence, primer slippage is a likely cause.

## Conclusions

This study shows that high primer degeneracy, when combined with low sequence diversity in the primer binding sites and flanking regions, can lead to slippage, producing sequences that are a few bp shorter or longer than the expected amplicon length. As this effect is template specific, its extent can vary substantially, even among closely related species in a particular sample. This variation can create analytical complexity, especially when clustering algorithms consider flanking regions. Importantly, primer slippage can be mitigated by repositioning primers to more heterogeneous binding sites and by considering their flanking regions when designing primer sets.

## Electronic supplementary material

Scripts S1

Fig S1

Table S1
