# Supplementary material for: Slippage of degenerate primers can cause variation in amplicon length
Source: Sci Rep. 2018 Jul 20;8:10999. doi: 10.1038/s41598-018-29364-z (PMC6054607; doi:10.1038/s41598-018-29364-z)
Supplement: Supplementary file 2 — Fig S1 [file 41598_2018_29364_MOESM2_ESM.pdf]

Supplementary Info: Slippage of degenerate primers can cause variation in amplicon length

Vasco Elbrecht, Paul D.N. Hebert, Dirk Steinke

Fig. S1: Plots of length variation for six additional primers.

Table S1: Raw length distribution data and number of sequences used for each taxon and primer. (Uploaded separately)

Scripts S1: R scripts used to analyze primer length distribution. (Uploaded separately)

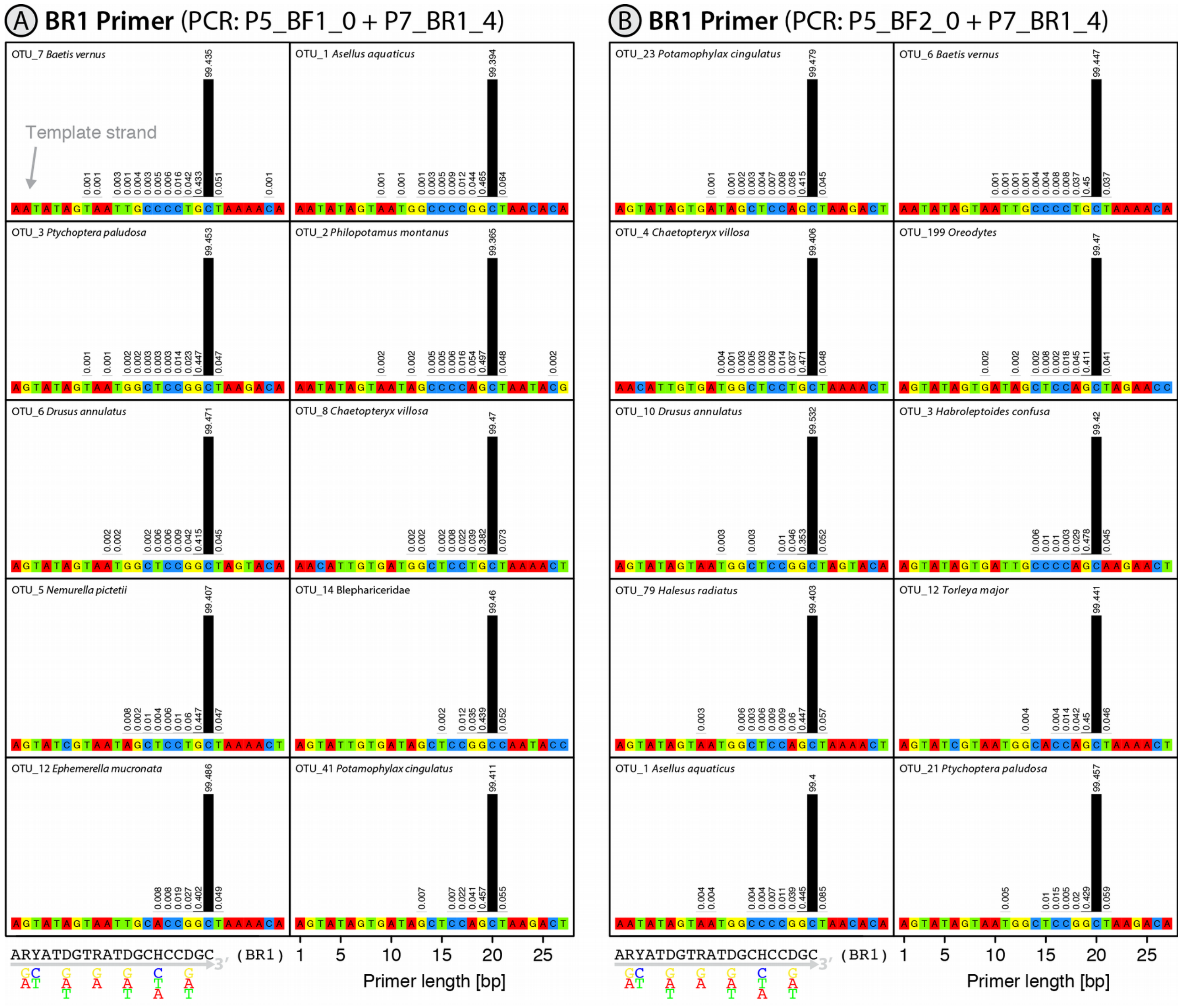

Figure S1: Plot of primer binding sites and bar plots depicting length of utilised primers for the 10 most abundant OTUs in mock sample B (sequence data from Elbrecht & Leese 2017). The presence and read abundance for different taxa can vary based on the primer set used, thus while amplifying the same mock samples, the order of and taxa the 10 most abundant specimens is not identical between plots as OTUs are sorted by read abundance. The sample was amplified with the P5_BF1_0 + P7_BR1_4 and P5_BF2_0 + P7_BR1_4 primer set, and the length distribution of the incorporated primers is shown for the BR1 primer (A and B). The percentage of amplicons that incorporated a particular length are shown above each bar.

Additional plots for further primer combinations on the next 2 pages (C, D, E).

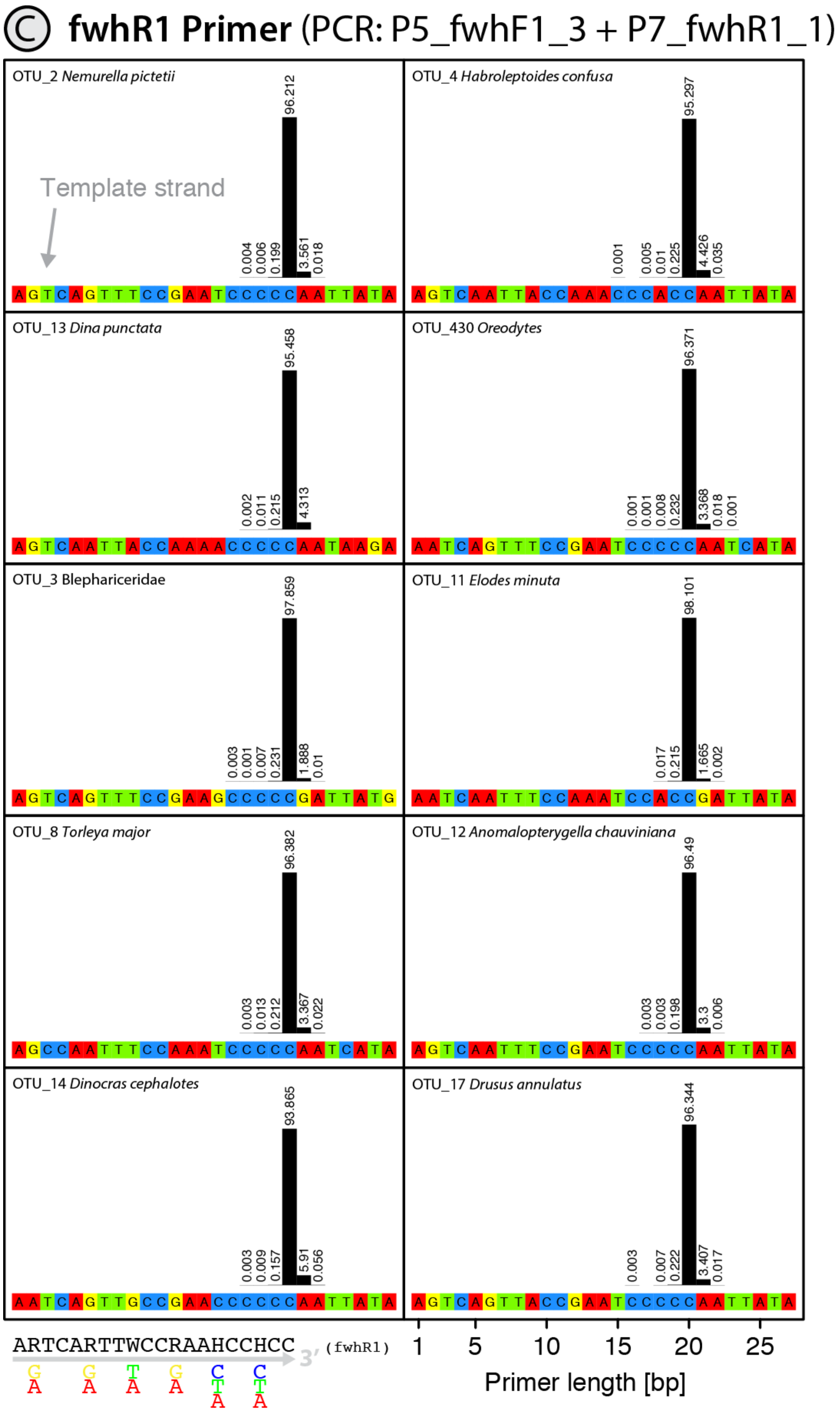

C: Length distribution of the fwhR1 primer (Sample B, P5_fwhF1_3 + P7_fwhR1_1, Vamos et al. 2017). The fwhF1 primer was not analyzed, as no data about the primer binding site was available (as it binds in the LCO1490 primer binding region which was not sequenced).

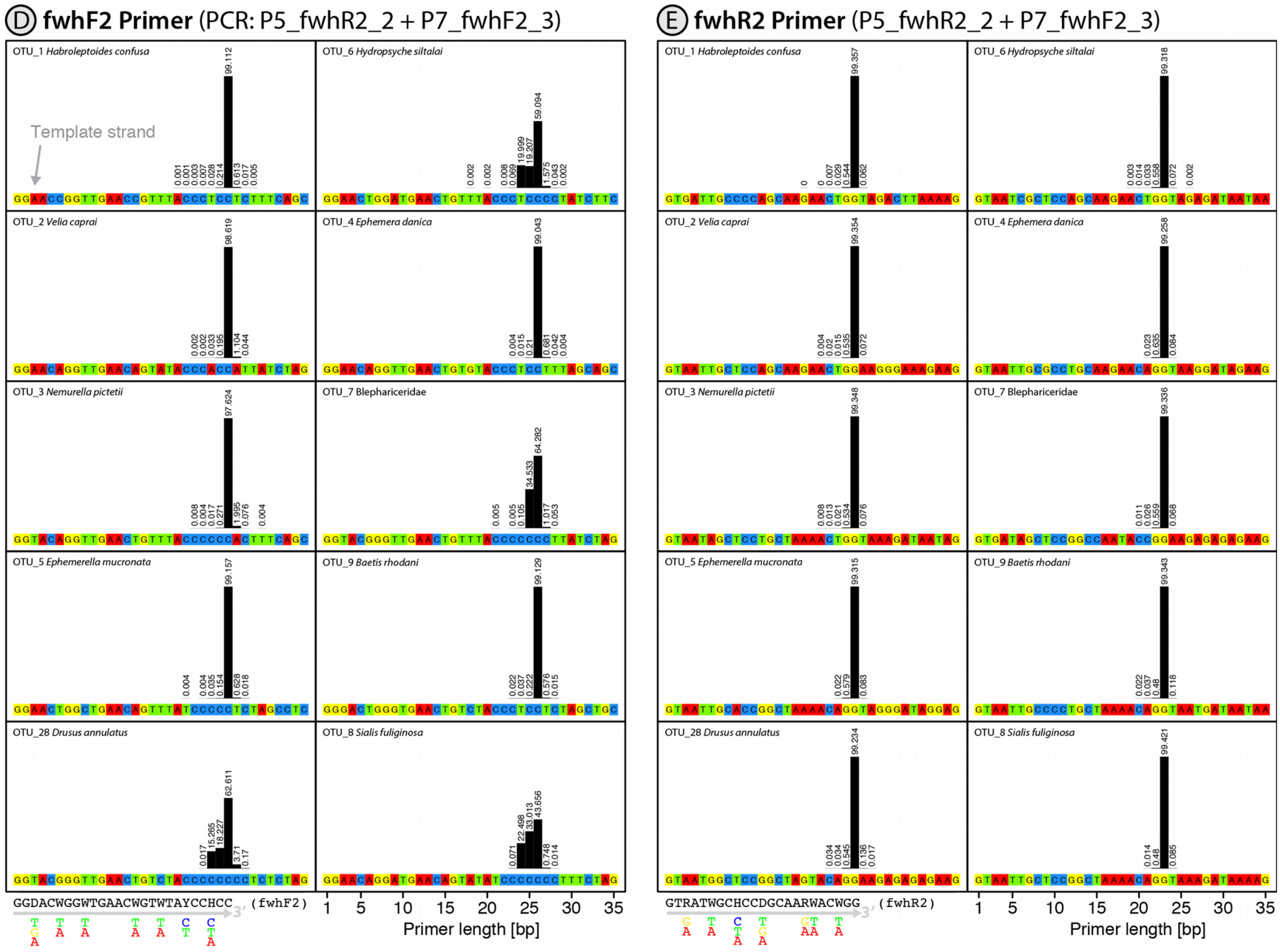

D & E: Length distribution of the fwhF2 and fwhR2 primer (Sample B, P5_fwhR2_2 + P7_fwhF2_3, Vamos et al. 2017).

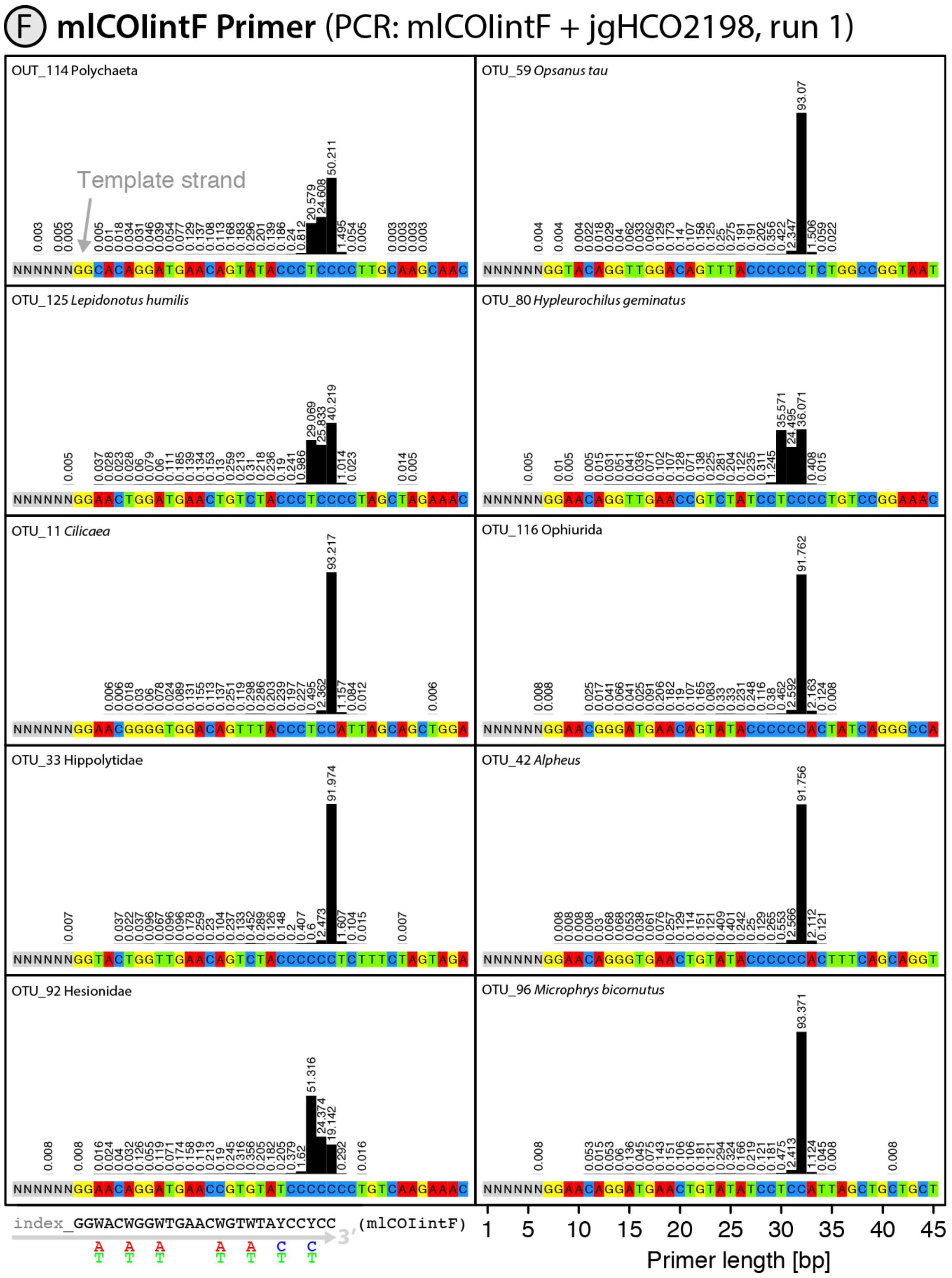

F: Length distribution of the mlCOIintF primer (marine invertebrate sample from Leray & Knowlton 2017, mlCOIintF + jgHCO, run 1). The jgHCO primer was not analyzed, as no data about the primer binding site was available (as it binds in the HCO2198 primer binding region which was not sequenced).
